# Ticagrelor potentiates adenosine-induced stimulation of neutrophil chemotaxis and phagocytosis

**DOI:** 10.1016/j.vph.2015.02.006

**Published:** 2015-08

**Authors:** Khalaf F. Alsharif, Mark R. Thomas, Heather M. Judge, Haroon Khan, Lynne R. Prince, Ian Sabroe, Victoria C. Ridger, Robert F. Storey

**Affiliations:** a Department of Cardiovascular Science, University of Sheffield, Beech Hill Road, Sheffield S10 2RX, UK; b Department of Infection and Immunity, University of Sheffield, Beech Hill Road, Sheffield S10 2RX, UK

**Keywords:** Adenosine, Dipyridamole, Erythrocytes, Ticagrelor, Neutrophils

## Abstract

In the PLATO study, ticagrelor was associated with fewer pulmonary infections and subsequent deaths than clopidogrel. Neutrophils are a first-line defence against bacterial lung infection; ticagrelor inhibits cellular uptake of adenosine, a known regulator of neutrophil chemotaxis and phagocytosis. We assessed whether the inhibition of adenosine uptake by ticagrelor influences neutrophil chemotaxis and phagocytosis. Neutrophils and erythrocytes were isolated from healthy volunteers. Concentration-dependent effects of adenosine on IL-8-induced neutrophil chemotaxis were investigated and the involved receptors identified using adenosine receptor antagonists. The modulatory effects of ticagrelor on adenosine-mediated changes in neutrophil chemotaxis and phagocytosis of *Streptococcus pneumoniae* were determined in the presence of erythrocytes to replicate physiological conditions of cellular adenosine uptake. Low-concentration adenosine (10^− 8^ M) significantly increased IL-8-induced neutrophil chemotaxis (% neutrophil chemotaxis: adenosine 28.7% ± 4.4 vs. control 22.6% ± 2.4; p < 0.01) by acting on the high-affinity A_1_ receptor. Erythrocytes attenuated the effect of adenosine, although this was preserved by ticagrelor and dipyridamole (another inhibitor of adenosine uptake) but not by control or by cangrelor. Similarly, in the presence of erythrocytes, a low concentration of adenosine (10^− 8^ M) significantly increased neutrophil phagocytic index compared to control when ticagrelor was present (37.6 ± 6.6 vs. 28.0 ± 6.6; p = 0.028) but had no effect in the absence of ticagrelor. We therefore conclude that the inhibition of cellular adenosine reuptake by ticagrelor potentiates the effects of a nanomolar concentration of adenosine on neutrophil chemotaxis and phagocytosis. This represents a potential mechanism by which ticagrelor could influence host defence against bacterial lung infection.

## Graphical abstract

**Figure:**
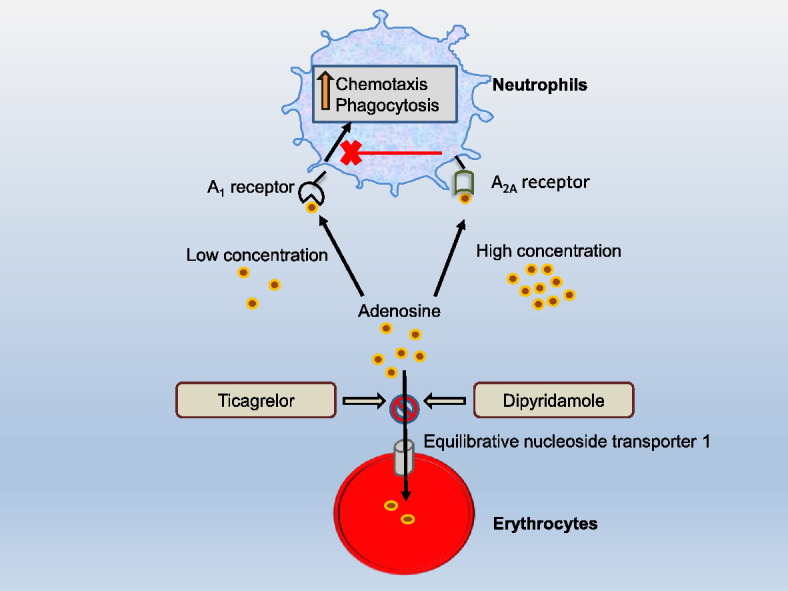

## 1 Introduction

Ticagrelor is a novel class of antiplatelet medication that inhibits platelet P2Y_12_ receptors and also inhibits cellular uptake of adenosine by inhibiting equilibrative nucleoside transporter 1 (ENT1) [1,2]. In the PLATelet inhibition and patient Outcomes (PLATO) study, ticagrelor reduced the incidence of adverse cardiovascular events in patients with acute coronary syndromes (ACS) compared to clopidogrel [3]. Accounting for this reduction in cardiovascular events, ticagrelor provides more potent and consistent P2Y_12_ inhibition than clopidogrel, leading to a greater antithrombotic effect [4,5]. In addition, by inhibiting cellular uptake of adenosine, ticagrelor increases plasma levels of adenosine in ACS patients [6]. Clinically relevant effects of this mechanism have been suggested by potentiation of the effect of adenosine on coronary blood flow and dyspnoea by ticagrelor [7,8]. Additional cardiovascular effects of this mechanism include adenosine-mediated limitation of myocardial infarct size [9] and adenosine-mediated inhibition of platelet aggregation by ticagrelor [10].

The reduction in all-cause mortality with ticagrelor compared to clopidogrel in the PLATO study (HR 0.78; 95% CI 0.69–0.89; p < 0.001) was unexpected on the basis of previous studies, raising the important possibility that beneficial non-cardiovascular pleiotropic effects may have contributed to this mortality reduction. Further analysis of the PLATO study revealed that ticagrelor was also associated with fewer pulmonary infections and fewer deaths related to infection and sepsis than clopidogrel [11–13]. The PLATO study demonstrated that levels of inflammatory markers and neutrophil counts were unexpectedly slightly, but significantly, higher in the ticagrelor group compared to the clopidogrel group [11]. This further suggests a differential effect of the two medications on innate immune responses.

By inhibiting cellular uptake of adenosine, ticagrelor increases extracellular levels of adenosine [6], which is known to be a major mediator of inflammation and innate immunity [14]. Adenosine is a degradation product of ATP that is released during conditions of cellular stress, such as ischaemia and inflammation. At low concentrations, adenosine signals via A_1_ and A_3_ receptors [15]. This primes the innate immune system to respond to tissue damage by upregulating pro-inflammatory functions of neutrophils and macrophages, such as chemotaxis (the directional migration of neutrophils towards chemical stimuli) and phagocytosis [15]. Conversely, at higher concentrations, adenosine acts via lower affinity A_2A_ and A_2B_ receptors to limit innate immune activation to prevent excessive collateral tissue damage [15]. Activation of A_2A_ and A_2B_ receptors has a wide range of anti-inflammatory effects, including downregulation of phagocytosis, chemotaxis and cytokine production in neutrophils [15]. Adenosine is rapidly taken up into erythrocytes by ENT1, resulting in a half-life of less than 10 s in the blood due to the great number of erythrocytes. ENT1 is almost ubiquitously expressed to a varying degree by human cells and therefore also regulates extracellular levels of adenosine within tissue [16].

We therefore hypothesised that ticagrelor would potentiate the effect of adenosine on neutrophil chemotaxis and phagocytosis by inhibiting cellular uptake of adenosine. Dipyridamole is a known inhibitor of adenosine uptake [17] and was therefore used as a positive control.

## 2 Methods

### 2.1 Neutrophil and erythrocyte isolation

Neutrophils were isolated from human peripheral blood, based on a previously described method [18]. Briefly, 8.9 ml venous blood was collected from healthy volunteers and immediately transferred to tubes containing 1.1 ml of sodium citrate (3.8%; Martindale Pharmaceuticals, UK). The anticoagulated blood was centrifuged at 300 ×*g* for 20 min to pellet the leukocytes and platelet-rich plasma was discarded. Erythrocytes were sedimented using 6% dextran (Sigma-Aldrich, UK) for 30 min at room temperature. Leucocyte-rich plasma was withdrawn, layered gently over 15 ml Histopaque 1077 (Sigma-Aldrich, UK) and centrifuged (400 ×*g*, 25 min). Supernatant was discarded and the pellet subjected to hypotonic lysis (0.2% NaCl) to lyse residual erythrocytes and then hypertonic rescue buffer (1.6% NaCl supplemented with 0.1% glucose). The cell suspension was centrifuged (300 ×*g*, 7 min) and resuspended in RPMI buffer (Life Technologies Ltd., UK).

In parallel with the above, 3 ml of blood from the healthy volunteers was immediately transferred to tubes containing sodium citrate 3.8%. Platelet-rich plasma was discarded after centrifugation (300 ×*g*, 20 min). The erythrocyte-rich leukocyte pellet was resuspended in buffer, layered over 15 ml Histopaque 1077 and centrifuged (400 ×*g*, 25 min). In order to avoid blocking of the pores of the chemotaxis assay, the erythrocyte:neutrophil ratio was altered, by increasing the neutrophil concentration using erythrocyte-free isolated neutrophils, to give a final concentration of 2 × 10^6^ neutrophils ml^− 1^.

### 2.2 Transmigration of neutrophils in vitro

A 96-well chemotaxis chamber (Neuro Probe, Inc., Gaithersburg, MD) was used to measure neutrophil chemotaxis, as previously described [18]. Briefly, the lower wells of a microplate were loaded with 30 μl of control medium (RPMI) containing different concentrations of IL-8 (10^− 10^–10^− 7^ M), a potent CXC chemokine that is used to induce human neutrophil chemotaxis in vitro. To assess the chemotactic response of neutrophils to adenosine, 30 μl of adenosine (10^− 8^–10^− 5^ M) was added to the lower wells. Assays were performed in duplicate. 30 μl of neutrophils (2 × 10^6^ ml^− 1^) ± adenosine ± inhibitors ± erythrocytes was placed directly onto the 5 μm filter membrane. The chemotaxis chamber was incubated for 30 min (37 °C, 5% CO_2_) as described previously [19]. The number of cells that had migrated into the lower chamber was calculated as a percentage of the total number of cells added to the filter.

### 2.3 Adenosine and adenosine receptor or reuptake antagonists

Isolated neutrophils were incubated with freshly prepared adenosine (Sigma-Aldrich, UK) at a range of concentrations from 10^− 11^ M to 10^− 5^ M immediately prior to studying chemotaxis. Further experiments were performed following preincubation with adenosine receptor antagonists (10^− 7^ M) DPCPX (A_1_ antagonist; Sigma-Aldrich, UK), SCH58261 (A_2A_ antagonist; Sigma-Aldrich, UK) or MRS 1334 (A_3_ antagonist; Tocris Bioscience, UK) for 20 min (37 °C, 5% CO_2_).

In some experiments neutrophils, either in the presence or in the absence of erythrocytes and/or adenosine (10^− 8^ M or 10^− 5^ M), were incubated with 10^− 5^ or 10^− 6^ M of either cangrelor (gift from The Medicines Company, New Jersey, USA), ticagrelor (Sequoia Research Products Limited, UK) or dipyridamole (Sigma-Aldrich, UK) for 20 min prior to the chemotaxis assay.

### 2.4 Neutrophil phagocytosis

Neutrophils (2.5 × 10^6^ ml^− 1^), either in the presence or in the absence of erythrocytes and/or adenosine (10^− 8^ M or 10^− 5^ M), were resuspended in RPMI with 10% foetal bovine serum with and without ticagrelor (10^− 5^ M) in a final volume of 100 μl in a 96-well plate. Assays were performed in duplicate. To allow visualisation of neutrophil phagocytosis by microscopy, it was necessary to dilute the erythrocytes, which were therefore resuspended at 12.5 × 10^6^ ml^− 1^. Heat-killed, opsonized *Streptococcus pneumoniae* was added to achieve a multiplicity of infection (MOI) of 20 and incubated for 30 min (37 °C, 5% CO_2_). Cytocentrifuge slides were prepared from the cell suspension using a Cytospin machine (Shandon, Thermo Scientific, Waltham, MA) and stained with modified Giemsa based stains (Differentiation-Quik, Reagena, Toivala, Findland). The percentage of neutrophils containing phagocytosed *S. pneumoniae* was determined by assessment of 300 neutrophils by light microscopy. Neutrophil phagocytic index was then determined using the following formula: (total number of engulfed bacteria / total number of counted neutrophils) × (number of neutrophils containing engulfed bacteria / total number of counted neutrophils) [20].

### 2.5 Statistical methods

Results are presented as mean ± SEM. Assuming a mean neutrophil chemotaxis rate of 20% with SD of 3.0%, 6 repeat experiments were required to provide 80% power to detect a 25% relative increase in neutrophil chemotaxis in response to adenosine with α of 0.05. Statistical analyses were performed using GraphPad Prism version 6.04 (GraphPad Software Inc., La Jolla, CA). Analysis of variance was used for statistical significance followed by Dunnett's test to compare the treated groups with vehicle control or Bonferroni's test to compare selected groups. p value < 0.05 was considered significant.

## 3 Results

### 3.1 Effect of adenosine on neutrophil chemotaxis

**Fig. 1:**
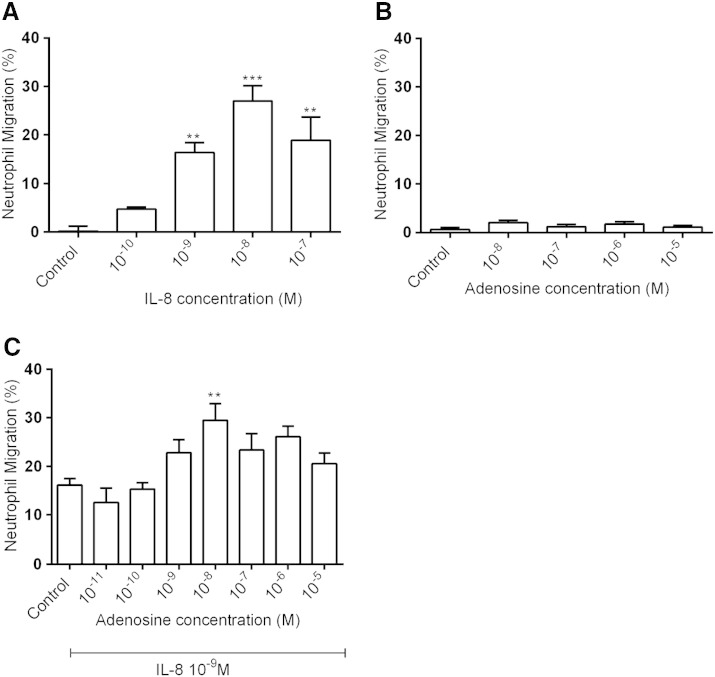
Effects of IL-8 and adenosine on neutrophil chemotaxis. Chemotactic response of neutrophils to increasing concentrations of IL-8 (A; n = 4) or adenosine (B; n = 4). The effect of increasing concentrations of adenosine on neutrophil chemotaxis induced by IL-8 10^− 9^ M (C; n = 8). The number of neutrophils that migrated over 30 min was counted and results expressed as a percentage of the total number of neutrophils added to the filter membranes of chemotaxis chambers. Results are presented as mean ± SEM and analysed for statistical significance using one-way analysis of variance followed by Dunnett's *t*-test. **p < 0.01 and ***p < 0.001 compared to control.

There was a maximal response of isolated human neutrophils to IL-8 at a concentration of 10^− 8^ M with lower response at higher concentration (Fig. 1A), as previously described [18]. A sub-maximal concentration (10^− 9^ M) was used for all subsequent experiments to investigate any potential increase or decrease in chemotaxis caused by adenosine. Next, we investigated whether adenosine acts as a chemoattractant for neutrophils in vitro. When adenosine (10^− 8^–10^− 5^ M) was added to the lower wells of the chemotaxis assay chamber, there was no significant effect on the migratory behaviour of the isolated neutrophils compared to RPMI control (Fig. 1B). We then tested the effect of the presence of increasing concentrations of adenosine on the neutrophil response to IL-8 (10^− 9^ M). The presence of adenosine at a concentration of 10^− 8^ M induced a significant increase in neutrophil chemotaxis (Fig. 1C) and was therefore used in subsequent experiments.

### 3.2 Identifying the role of adenosine receptors in neutrophil chemotaxis

**Fig. 2:**
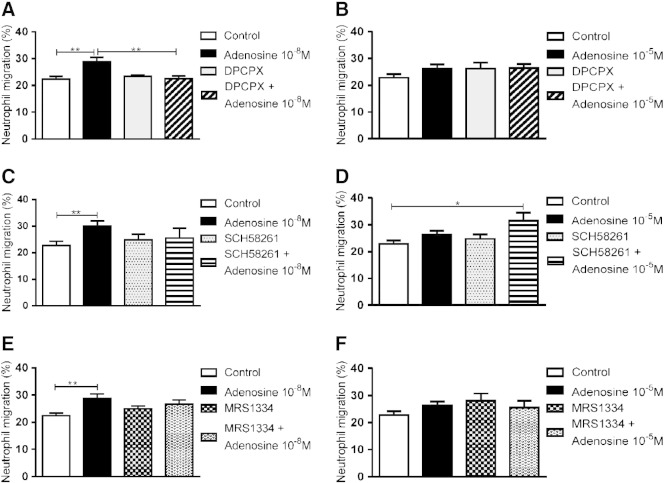
Effect of adenosine receptor antagonists on neutrophil chemotaxis in the presence of adenosine. The effect of the A_1_ antagonist DPCPX 10^− 7^ M (A and B), the A_2_ antagonist SCH58261 10^− 7^ M (C and D) and the A_3_ antagonist MRS 1334 10^− 7^ M (E and F) on neutrophil migration to IL-8 10^− 9^ M over 30 min in the presence or absence of adenosine 10^− 8^ M (A, C and E; n = 6) or adenosine 10^− 5^ M (B, D and F; n = 7). All inhibitors were assessed within the same experiment but are divided into different panels for clarity. Results are presented as mean ± SEM and analysed for statistical significance using one-way analysis of variance followed by Bonferroni's test for multiple comparisons. *p < 0.05 and **p < 0.01.

DPCPX (10^− 7^ M), a specific antagonist of the A_1_ receptor [21], blocked the augmentation of IL-8-induced neutrophil chemotaxis by adenosine (10^− 8^ M; Fig. 2A) but had no effect in the presence of the higher concentration of adenosine (10^− 5^ M; Fig. 2B). Conversely, the treatment of neutrophils with SCH58261 (10^− 7^ M), a specific antagonist of the A_2A_ receptor [22], had no effect in the presence of 10^− 8^ M adenosine (Fig. 2C) but, in the presence of a higher concentration of adenosine (10^− 5^ M), significantly increased neutrophil chemotaxis towards IL-8 (Fig. 2D). The A_3_ receptor antagonist MRS 1334 (10^− 7^ M) did not affect neutrophil chemotaxis in the presence of either 10^− 8^ M or 10^− 5^ M adenosine (Fig. 2E and F).

### 3.3 Erythrocytes attenuate the effect of adenosine on neutrophil chemotaxis

**Fig. 3:**
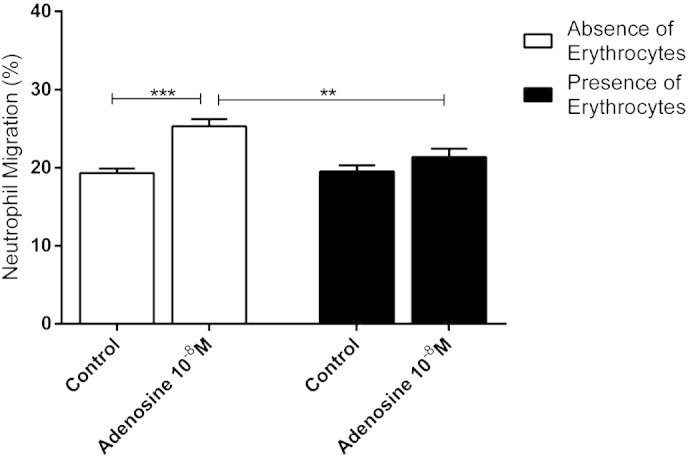
Effect of erythrocytes on the response to adenosine. Chemotactic response of neutrophils to IL-8 10^− 9^ M, in the presence of adenosine 10^− 8^ M and in the absence (white columns) or presence (black columns) of erythrocytes (n = 14). Results are presented as mean ± SEM and analysed for statistical significance using one-way analysis of variance followed by Bonferroni's test for multiple comparisons. **p < 0.01 and *** < 0.001.

To explore the effects of physiological cellular uptake of adenosine, the effect of adding erythrocytes to the neutrophil suspension was assessed. Whereas adenosine (10^− 8^ M) significantly potentiated neutrophil chemotaxis towards IL-8 in the absence of erythrocytes, this effect was not seen in the presence of erythrocytes (Fig. 3).

### 3.4 Ticagrelor and dipyridamole enhance neutrophil chemotaxis by inhibiting cellular uptake of adenosine

**Fig. 4:**
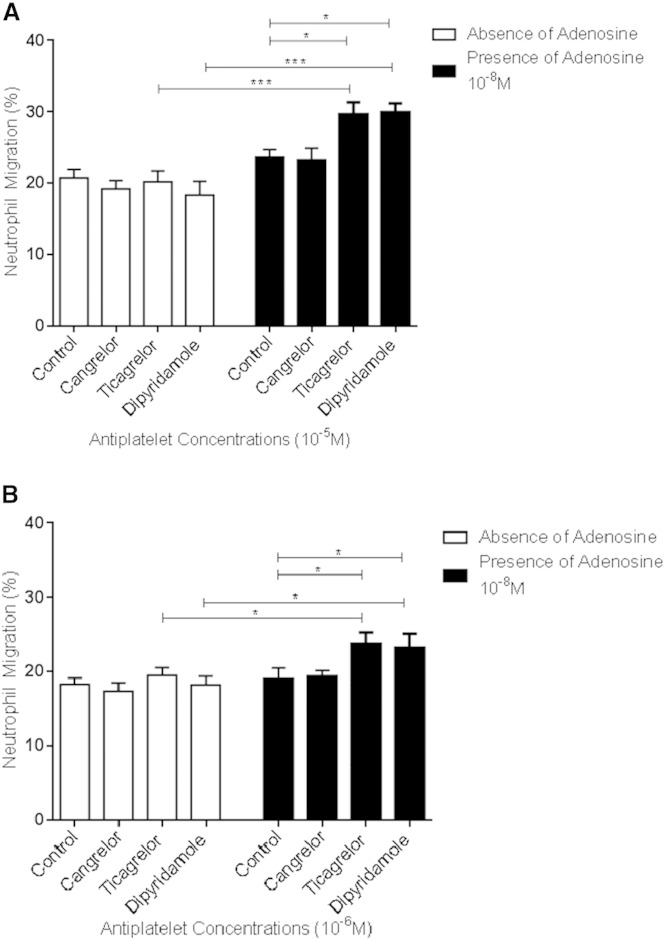
Effects of cangrelor, ticagrelor and dipyridamole on neutrophil chemotaxis in the presence of erythrocytes and the absence or the presence of adenosine. Chemotactic response of neutrophils to IL-8 10^− 9^ M, in the presence of erythrocytes and without (white columns) or with (black columns) addition of adenosine 10^− 8^ M, showing the effects of cangrelor, ticagrelor and dipyridamole all at concentrations of either (A) 10^− 5^ M or (B) 10^− 6^ M, compared to vehicle control (n = 7). Results are presented as mean ± SEM and analysed for statistical significance using one-way analysis of variance followed by Bonferroni's test for multiple comparisons.*p < 0.05, ***p < 0.001 and **** < 0.0001.

None of the platelet inhibitors tested (cangrelor, ticagrelor and dipyridamole; 10^− 5^ M) altered IL-8-induced neutrophil chemotaxis in the presence of erythrocytes and in the absence of adenosine (Fig. 4). However, in the presence of both erythrocytes and adenosine, ticagrelor and dipyridamole significantly increased IL-8-induced neutrophil chemotaxis (Fig. 4). No such effect was seen with cangrelor, a P2Y_12_ inhibitor that has no effect on cellular adenosine uptake [2].

### 3.5 Ticagrelor potentiates neutrophil phagocytosis induced by low concentrations of adenosine

**Fig. 5:**
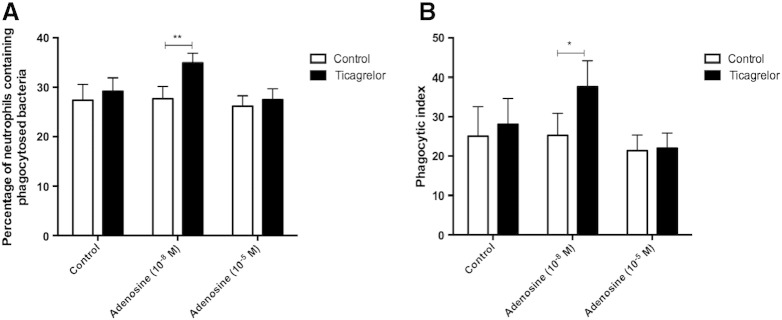
Effect of ticagrelor on changes in neutrophil phagocytosis induced by low and high concentrations of adenosine in the presence of erythrocytes. Effect of ticagrelor (10^− 5^ M) on changes in neutrophil phagocytosis of *S. pneumoniae*, determined by percentage of neutrophils containing phagocytosed *S. pneumoniae* (A) and phagocytic index (B), induced by 10^− 8^ M and 10^− 5^ M adenosine in the presence of erythrocytes (n = 8). Results are expressed as mean ± SEM and analysed for statistical significance using two-way ANOVA followed by Bonferroni's test for multiple comparisons. *p < 0.05, **p < 0.01.

In the presence of erythrocytes, a low concentration of adenosine (10^− 8^ M) significantly increased the percentage of neutrophils containing phagocytosed *S. pneumoniae* (35.0% ± 1.9 vs. 27.7% ± 2.5; p = 0.0029) (Fig. 5A) and neutrophil phagocytic index compared to control (37.6 ± 6.6 vs. 28.0 ± 6.6; p = 0.028) (Fig. 5B) when ticagrelor (10^− 5^ M) was present. In contrast, in the absence of ticagrelor, low concentration adenosine (10^− 8^) had no effect on percentage of neutrophils containing phagocytosed *S. pneumoniae* (27.7% ± 2.5 vs. 27.4% ± 3.2; p > 0.05) (Fig. 5A) or phagocytic index (25.3 ± 5.6 vs. 25.1 ± 7.5; p > 0.05) (Fig. 5B). A higher concentration of adenosine (10^− 5^ M) did not affect neutrophil phagocytosis, likely due to the activation of lower-affinity A_2A_ receptors.

**Fig. 6:**
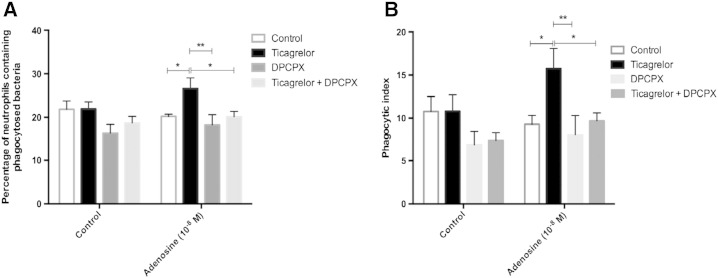
Interaction between ticagrelor and the A1 receptor antagonist DPCPX. Effect of ticagrelor (10^− 5^ M) and DPCPX (10^− 7^ M) on neutrophil phagocytosis of *S. pneumoniae* determined by percentage of neutrophils containing phagocytosed *S. pneumoniae* (A) and phagocytic index (B), induced by 10^− 8^ M adenosine in the presence of erythrocytes (n = 5). Results are expressed as mean ± SEM and analysed for statistical significance using two-way ANOVA followed by Bonferroni's test for multiple comparisons. *p < 0.05, **p < 0.01.

The potentiation of adenosine-mediated neutrophil phagocytosis caused by ticagrelor was A_1_ receptor dependent (Fig. 6). In the presence of erythrocytes, DPCPX (an A_1_ receptor antagonist) significantly inhibited the effect of ticagrelor on potentiating the stimulatory effect of low-concentration adenosine (10^− 8^ M) on the percentage of neutrophils containing phagocytosed bacteria (p < 0.01) (Fig. 6A) and the neutrophil phagocytic index (p < 0.01) (Fig. 6B).

## 4 Discussion

We have shown for the first time that adenosine reuptake inhibition with ticagrelor potentiates the increases in neutrophil chemotaxis and phagocytosis mediated by adenosine in vitro. It has now been consistently demonstrated that ticagrelor inhibits cellular uptake of adenosine [1,2] and we have also confirmed this in our laboratory (data not shown). This has been shown to increase plasma levels of adenosine in patients with ACS [6] and so supports the hypothesis that ticagrelor treatment might have relevant effects on neutrophil function in vivo. Four different adenosine receptors exist, which are activated at different concentrations of adenosine and expressed on a wide range of different cell types [23]. The resultant pleiotropic effects of this mechanism are therefore complex. This study demonstrates that this mechanism affects important neutrophil responses, in addition to previously described cardiovascular effects [7–10]. Comparison with dipyridamole suggests that this is a class-effect of ENT1 inhibitors.

To investigate whether adenosine reuptake inhibition by ticagrelor might influence leukocyte function, we first identified the appropriate concentration of IL-8 to induce neutrophil chemotaxis. We found that IL-8 was able to induce significant chemotaxis with a maximum effect at a concentration of 10^− 8^ M, consistent with previous studies [18,24]. Although adenosine itself was not able to act as a chemoattractant for neutrophils, a nanomolar concentration of adenosine was found to potentiate IL-8-induced neutrophil chemotaxis with loss of this effect at micromolar concentrations. This supports previous work suggesting that the lower concentrations of adenosine promote neutrophil chemotaxis, whereas high concentrations of adenosine inhibit neutrophil chemotaxis [25]. A similar result was also observed by adding adenosine (10^− 9^ M to 10^− 6^ M) in the lower wells with fMLP in a chemotaxis assay [26]. In addition, adenosine had a similar effect on human monocytes [27]. Analogous findings have been reported also for ATP, from which adenosine is derived by intracellular and extracellular breakdown [26,28].

To explore the function of different adenosine receptors in neutrophil chemotaxis, specific adenosine receptor antagonists were used in the presence of high and low concentrations of adenosine. Our results revealed that the low concentration of adenosine stimulates neutrophil chemotaxis through the A_1_ receptor. In contrast, activation of the A_2A_ receptor by a high concentration of adenosine attenuates neutrophil chemotaxis in response to IL-8. Our findings are consistent with previous studies indicating that the high affinity A_1_ receptor promotes neutrophil chemotaxis, whereas the lower affinity A_2A_ receptor limits neutrophil chemotaxis [25,27,29]. This likely reflects the different signalling pathways linked to these receptors: A_1_ is G_i/o_-coupled and the occupancy of A_1_ diminishes cAMP accumulation, whereas A_2A_ is G_s_-coupled and the binding of adenosine to the A_2A_ receptor increases the formation of cAMP [29]. The role of A_3_ receptors in neutrophil chemotaxis is more controversial. Some studies showed that A_3_ receptors enhance neutrophil chemotaxis [26,30], whereas other studies, like our own, have not confirmed this [31,32]. Our findings are also consistent with previous studies that show that nanomolar concentrations of adenosine stimulate neutrophil phagocytosis by acting on high-affinity A_1_ receptors [33,34]. Previous studies have demonstrated an inhibitory effect of micromolar concentrations of adenosine on neutrophil phagocytosis mediated by A_2A_ receptors [33,34], although our results demonstrate a more neutral effect.

Although no study has focused on the effect of ticagrelor as an adenosine uptake inhibitor on neutrophil function, dipyridamole has been found to exert beneficial pleiotropic effects secondary to an action on neutrophils. For example, preoperative treatment with dipyridamole for patients who undergo coronary artery bypass graft inhibited neutrophil superoxide anion generation and neutrophil adhesion to endothelial cells [35]. These researchers proposed that this mechanism is mediated by increased adenosine levels. Another study suggested that dipyridamole enhanced the inhibitory effects of adenosine, which, in turn, reduced the effect of fMLP-activated neutrophil hydrogen peroxide (H_2_O_2_) production [27].

Our results demonstrate how adenosine uptake inhibition by dipyridamole and ticagrelor can preserve the extracellular concentration of adenosine in the presence of erythrocytes, which in turn enhances neutrophil chemotaxis and phagocytosis through stimulation of A_1_ receptors. Although ticagrelor has been shown to induce ATP release from human erythrocytes in vitro, which is subsequently degraded to adenosine [36], our results did not demonstrate any effect via this mechanism on neutrophil recruitment, since there was no effect when ticagrelor was combined with erythrocytes and neutrophils in the absence of added adenosine.

P2Y_12_ inhibitors reduce platelet-neutrophil aggregate formation and release of inflammatory-mediators from platelet α-granules [37]. Platelet–leukocyte aggregates are pro-inflammatory and may be harmful in conditions associated with excessive immune activation, such as sepsis and acute lung injury [38]. However, platelet–neutrophil aggregates are primed for phagocytosis and intracellular killing [38] and it is therefore feasible that inhibiting their formation may hinder initial resolution of bacterial infection. It is possible that this is to some extent counter-balanced by ticagrelor potentiating A_1_-mediated neutrophil chemotaxis and phagocytosis at low levels of adenosine, such as may occur at the early stages of infection, however. In conditions such as sepsis, adenosine is present at higher concentrations and acts on A_2A_ and A_2B_ receptors to dampen excessive inflammation [39]. Therefore, in contrast, potentiating the effect of adenosine in sepsis might have anti-inflammatory effects. Taken together, these findings provide mechanisms that may be relevant to the observation of fewer pulmonary infections and fewer deaths following pulmonary infections and sepsis during treatment with ticagrelor compared to clopidogrel in the PLATO study.

In conclusion, ticagrelor enhanced the stimulatory effect of a nanomolar concentration of adenosine on neutrophil chemotaxis and phagocytosis under physiological conditions of cellular adenosine uptake. Ticagrelor and dipyridamole had no direct effect on neutrophil recruitment and phagocytosis but were able to preserve the enhancing effect of adenosine in the presence of erythrocytes through the inhibition of adenosine reuptake. Further work is required to determine whether adenosine might mediate immunostimulatory effects of ticagrelor that could provide protection against pulmonary infection and whether there is an optimal level of ENT1 inhibition that maximises any such effects.

## Addendum

K. Al-Sharif, M. Thomas, V. Ridger, H. Judge and R. Storey designed the research. K. Al-Sharif performed the literature search and V. Ridger and R. Storey reviewed the articles for inclusion. K. Al-Sharif conducted the neutrophil chemotaxis experiments and M. Thomas conducted the neutrophil phagocytosis experiments. K. Al-Sharif and M. Thomas performed the statistical analysis and drafted the manuscript which was revised by R. Storey and V. Ridger. H. Khan designed and conducted experiments to confirm the effects of the antiplatelet drugs on adenosine uptake by erythrocytes. All authors critically reviewed and revised the manuscript and approved the final version.

## Disclosures

R. Storey declares institutional research grants, honoraria and/or consultancy fees from Accumetrics, AstraZeneca, Aspen, Correvio, Medscape, Merck, PlaqueTec, Regeneron, Roche, Sanofi Aventis, ThermoFisher Scientific and The Medicines Company. The other authors have no disclosures.
